# Seasonal Food Insecurity among Farm Workers in the Northern Cape, South Africa

**DOI:** 10.3390/nu11071535

**Published:** 2019-07-05

**Authors:** Stephen Devereux, Lauren Tavener-Smith

**Affiliations:** 1 Institute of Development Studies, University of Sussex, Brighton BN1 9RE, UK; 2 Institute for Social Development, University of the Western Cape, Cape Town 7535, South Africa; 3 Centre of Excellence in Food Security, University of the Western Cape, Cape Town 7535, South Africa

**Keywords:** food security, seasonality, farm workers, dietary diversity, coping strategies

## Abstract

Very little is known about seasonal hunger in South Africa, or about the food security and nutritional status of farm workers. This article identifies a pathway to seasonal hunger—through intra-annual fluctuations in agricultural employment and income—that is underanalyzed in the literature. We report on findings from a year-long data collection process, comprising baseline and endline surveys and monthly monitoring of three food security indicators, with a sample of 195 female farm workers in the Northern Cape province in South Africa. The three monthly monitoring indicators—the Household Food Insecurity Access Scale (HFIAS), Dietary Diversity Score (DDS), and Coping Strategies Index (CSI)—which measure different aspects of food insecurity, are analyzed to determine whether and to what extent food security fluctuates seasonally in our sample. HFIAS results show unambiguous evidence of seasonal food insecurity, with the highest prevalence (88 percent experiencing severe food insecurity) and severity during the low employment winter period, and lowest prevalence (49 percent) and severity during the summer harvest, which corresponds with relatively higher employment and earnings. The DDS results show evidence of highest dietary diversity during summer and the CSI results reveal the need to employ coping strategies to deal with intensified food insecurity during winter.

## 1. Introduction

Seasonal food insecurity has been well documented in Africa, notably in the semi-arid Horn [1,2] and the West African Sahel [3,4]. The annual hungry season or *soudure* is typically associated with smallholders working on family farms, whose main source of subsistence is own produced food. In areas with one rainy season and a single main harvest, the agricultural year divides into two distinct periods: postharvest, when food is relatively plentiful and cheap, and preharvest, when food is relatively scarce and expensive. The combination of depleted food granaries and rising market prices induces rationing, hunger and undernutrition in poor subsistence-oriented farming households [5,6].

In this paper we identify a different pathway to seasonal hunger, through intra-annual fluctuations in agricultural employment and income. To our knowledge, this pathway has not been extensively explored in the literature on seasonality and agriculture. Nor has seasonal hunger been identified as an issue in South Africa. We argue that farm workers employed in commercial agriculture in South Africa are affected by seasonal food insecurity and hunger through this second pathway. Fluctuations in employment and income mirror agricultural production cycles, but farm workers do not produce food for their family’s consumption. Most are employed in producing fruit (citrus, deciduous, and table or wine grapes) rather than subsistence crops (cereals, legumes, roots, and tubers), and they are paid very low wages. In 2017 the legislated minimum wage for farm workers was set at R128 (less than US$10) a day and R2779 (less than US$200) a month [7]. Apart from the minority who have permanent contracts, most farm workers are unemployed during the winter months. Women are less likely than men to have permanent contracts [7,8], which leaves them especially vulnerable to seasonal food insecurity.

Very little is known about the food security and nutrition status of farm workers in South Africa. It is often asserted that farm workers are more vulnerable than other categories of workers. “Farmworkers earn the lowest wages of all formally employed persons in South Africa, generally live in poverty and experience high levels of food insecurity” [9] (p. 29). While intuitively credible, such assertions are rarely backed up with rigorous empirical evidence. We identified only two studies of malnutrition among farm workers [10,11], and occasional mentions in other reports of farm workers’ experiences of food insecurity.

In the late 1990s, the Transition and Health during the Urbanisation of South Africans (THUSA) study identified farm workers as a highly vulnerable group, in terms of their health and nutrition status. Most significantly, “children living on farms are very vulnerable and more likely to be stunted and underweight than any other children” [10] (p. 1).

A small nutrition survey of farm workers on commercial farms in a northwest province in 2002 found that one in four children (25 percent, *n* = 241) were stunted and one in five (19 percent) were underweight. Two-thirds of men (68 percent, *n* = 67) and two in five women (40 percent, *n* = 54) had body mass index (BMI) below 21 kg/m^2^ [11]. The main driver of malnutrition in farm worker families was identified as poverty, which was not alleviated by government policies such as minimum wages. “While the introduction of minimum wages in 2003 might have improved the economic situation of farm workers, in practice this sometimes means that farm owners cut previous benefits such as housing subsidies and food rations” [11] (p. 835), leaving them no better off than before.

A report by Oxfam in 2014 estimated that 13 million or one in four South Africans experience hunger, due to poverty, unemployment or low paid-work, and high food prices. “In Western Cape, farm workers described how their food supplies were exhausted by mid-week, forcing them to skip meals on Thursdays until they got paid again on Friday. During this period they eat porridge twice a day” [12] (p. 13). Many farm workers lost their jobs after the farm workers’ strike of 2013 [13], when higher minimum wages were secured that resulted in improved food security for some but worsened food insecurity for others: “2013 was very bad after the owners dismissed us because of the strike … it is very difficult to put food on the table” (unemployed farm worker, Western Cape) [12] (p. 18).

Seasonal food insecurity may be rising in South Africa, because the agricultural labor force is shifting away from workers living on farms with permanent contracts, towards seasonal or casual workers living off farms with short-term contracts or no contracts at all [14,15]. Women have been disproportionately affected by this process of ‘casualization’ [8]. Seasonal and casual workers are more vulnerable than permanent workers to food insecurity because they are employed only during the agricultural season, often at below the legislated minimum wage rate for the sector [7]. In the grape farms of the Western Cape and Northern Cape, the season starts with preparing the vines in August–September, and ends with packing grapes after the harvest, in March–April. Even during the farming season, employment opportunities are irregular, uncertain and precarious. During the months of May, June, and July there is virtually no work or income for seasonal and casual workers, the majority of who are women who also work fewer hours and earn less on average than men [8]. For this reason, our survey sampled only female farm workers.

This article investigates the phenomenon of underemployment-driven seasonal hunger among farm workers in a relatively remote and marginalized province of South Africa. Our hypothesis is that because employment on commercial farms is seasonal and remuneration is low, incomes also vary seasonally and so, consequently, do farm workers’ experiences of food insecurity and hunger. We expect hunger to be lowest in the months of highest employment (December to March), which coincides with the summer harvest season. Within this period, however, we expect an increase in hunger in January coinciding with the post-Christmas period (when expenses are particularly high) and the due date for payment of annual school fees. In the postharvest autumn and winter seasons (April–August) we anticipate that food insecurity and hunger will be at their highest levels, due to inadequate household incomes associated with this period of limited employment opportunities.

This article is structured as follows. The next section describes the research design, primary data collection and sampling, and the four indicators of household food (in)security that were collected and analyzed. Section 3 presents our results: sample descriptives, seasonality descriptives (indicator by indicator), and multivariate analysis, using ordinary least squares regression. Finally, the Discussion section reflects on the consistencies and dissonances in the findings and identifies gaps for further research and implications for policy.

## 2. Materials and Methods

The Farm Worker Food Security (FWFS) study aimed to explore the prevalence—extent, severity, and seasonality—of food insecurity among farm workers in two provinces of South Africa. Results are reported for one province. Research was conducted under the auspices of the DST-NRF Centre of Excellence in Food Security. Ethical approval was secured from the Humanities and Social Sciences Research Ethics Committee (HSSREC) at the University of the Western Cape.

### 2.1. Methodology Design and Data Collection

A structured questionnaire was designed and administered to approximately 200 female farm workers in the Northern Cape province of South Africa, in September 2017 and again (with modifications) in October 2018. The questionnaire (44 questions) included modules on personal and household demographics (13 questions), sources of food and income (9 questions), food and non-food expenditure (2 question), access to social protection (10 questions), experiences of hunger (8 questions), and coping strategies (2 questions). The same respondents were monitored on a monthly basis from October 2017 to September 2018, thereby constituting a panel with 14 observations. The monthly questionnaires (22 questions) consisted of one food security measurement tool, which alternated each month, and also asked about the number of days worked and social grants received by household members during the preceding month.

Four self-reported indicators were collected to assess levels of and trends in food insecurity among farm worker households: household food adequacy, household food insecurity, dietary diversity, and coping strategies. Each of these indicators has a well-established tool—Months of Adequate Household Food Provisioning (MAHFP), the Household Food Insecurity Access Scale (HFIAS), the Dietary Diversity Index (DDI), and the Coping Strategies Index (CSI), respectively—which are introduced below.

The MAHFP was included in the initial survey and the final survey. The other three instruments were administered in rotation in a randomly decided sequence, following a three-monthly cycle that began in October 2017 and was repeated four times, ending in September 2018. This allowed one observation to be recorded from each household for each instrument every quarter, and one observation per instrument per season (see Table 1). However, due to the proximity of the final monitoring round to the final survey and concerns about respondent fatigue, the fourth CSI interview was not undertaken in September 2018 as planned, so no measure for food security in spring based on this indicator is available for reporting.

Data collection was conducted in collaboration with Women on Farms Project (WFP), a non-governmental organization (NGO) that works with women farm workers and farm dwellers in the Western Cape and Northern Cape provinces of South Africa. WFP identified 15 literate women in rural farming communities who assisted in codesigning, pilot testing, and refining the survey instrument. They were then trained to administer all the questionnaires, including the monthly monitoring instruments. Their familiarity with local people and the local context allowed them to access commercial farms and to gain the trust of farm workers. Each respondent was given an information sheet explaining the research project and was asked to sign a consent form before being interviewed.

Sampling was nonrandom and purposive. Data collection was undertaken in four commercial farming areas in the Northern Cape. Each enumerator was allocated an area and a batch of 14 questionnaires to complete. Enumerators were instructed not to interview more than two farm workers from any one farm, then to proceed to the next accessible farm or locality until they had conducted 14 interviews. After cleaning this resulted in a valid sample of 195, who were re-interviewed by the same team of enumerators throughout the following year. All interviews were conducted in Afrikaans, the first language of the respondents and the enumerators, then transcribed and translated into English by bilingual students at the University of the Western Cape.

### 2.2. Food Security Measurement

As noted, four complementary measures of household food (in)security were collected. All four indicators are self-reported and are based on recall. The recall period ranges from 24 hours (for dietary diversity (DDS)) to one month (food insecurity access (HFIAS) and coping strategies (CSI)) to one year (months of adequate food (MAHFP)).

#### 2.2.1. Months of Adequate Household Food Provisioning (MAHFP)

The hunger module of the initial and final household questionnaire included the Months of Adequate Household Food Provisioning (MAHFP) indicator. The MAHFP allows researchers to construct a profile of household food (in)security during the preceding year, by asking respondents to recall any months during which they did not have enough food to meet their family’s needs [16]. The number of months of inadequate food provisioning is simply summed (from 0 to 12 months) to estimate the severity of food insecurity at the level of individual households. The MAHFP can be used to map seasonal or cyclical fluctuations in food insecurity at community or regional level, and to disaggregate the extent of seasonal food insecurity faced by different groups of respondents [17].

#### 2.2.2. Household Food Insecurity Access Scale (HFIAS)

The HFIAS asks nine questions about household access to food and household members’ experiences and perceptions of hunger, during the preceding four weeks (see Table 2). Each question has an associated intensity: either ‘0’ (none), ‘1’ (rarely), ‘2’ (sometimes), or ‘3’ (often). Responses are aggregated into a score (range 0 to 27) which can be interpreted as representing a continuum of food insecurity.

The nine questions reflect intensifying food insecurity and are clustered into three themes: anxiety about access to food, inadequate quality of diet, and inadequate food consumption. “The method is based on the idea that the experience of food insecurity (access) causes predictable reactions and responses that can be captured and quantified through a survey and summarized in a scale” [18] (p. 1). In this sense, the HFIAS shares some similarities with the Coping Strategies Index.

Typically, analysis and reporting of HFIAS uses the actual score computed by adding up the weighted (by frequency) responses to the above questions as well as a categorical version of the scores. On the basis of the HFIAS score, a household can be categorized into one of four levels of household food insecurity: (1) food secure, (2) mildly food insecure, (3) moderately food insecure, and (4) severely food insecure [19]. Compiling the results over all households in the sample allows percentage estimates of food secure and food insecure households in this population to be calculated.

We have chosen to report on two variations in the way that the HFIAS indicator scores are transformed into categorical variables. The first uses the categorization of HFIAS into four categories of increasing food insecurity (access) as detailed in the HFIAS Indicator Guide v3 [18]. The four categories comprise the following.

- Food Secure: may report worrying or being anxious about household’s food supply, but only rarely. Otherwise the household does not experience any other conditions of inadequate food access.
- Mildly food insecure: household does not reduce food intake nor experience running out of food, going hungry, or not eating for a whole day and night, but does worry about not having enough food (sometimes or often) and/or eats less preferred foods (rarely, sometimes, or often), and/or (rarely) limits their food variety and eats food that they really do not like eating.
- Moderately food insecure: does not run out of food, go to bed hungry, or not eat for 24 hours, but does often experience having to reduce quality and diversity of diet and/or (sometimes or rarely) cuts back on the size and frequency of meals.
- Severely food insecure: Often has to reduce meal size and frequency, and has run out of food entirely, gone to bed hungry, or not eaten for a whole day and night.

The second categorization is based on a South African study by Chakona and Shackleton (2018) [19] who create the four food insecurity categories by setting HFIAS score value thresholds to segment the entire distribution of HFIAS scores. These thresholds are

- Food secure: HFIAS score = 0–1
- Mildly food insecure: HFIAS score = 2–7
- Moderately food insecure: HFIAS score = 8–11
- Severely food insecure: HFIAS score = 12–27

The choice to report two sets of food insecurity prevalence estimates is (i) to be consistent with the Indicator Guide and thus generate estimates that are comparable with other studies that use the HFIAS; (ii) to be consistent with a local study in order to populate the local empirical literature and generate estimates that are consistent with other local studies; and (iii) to show how different categorizations lead to very different estimates of food insecurity (access) prevalence.

#### 2.2.3. Dietary Diversity Score (DDS)

A diversified diet is a simple but robust indicator of household food security, and is empirically correlated with higher levels of food consumption: “a 1 percent increase in dietary diversity is associated with a 1 percent increase in per capita consumption” [20] (p. iii). The dietary diversity score (DDS) is derived simply by classifying all food items into food groups, and adding up the number of items from discrete food groups that were consumed by household members during the previous 24 h.

Swindale and Bilinsky (2006) [21] propose the following 12 food groups: cereals, root and tubers, vegetables, fruits, meat, poultry, offal, eggs, fish and seafood, pulses/legumes/nuts, milk and milk products, oil/fats, sugar/honey, and miscellaneous. A slightly different configuration of 12 groups is preferred by FAO (2010) [22]: cereals, tubers and roots, legumes, nuts and seeds, milk and milk products, eggs, fish, meat, oils and fats, vegetables, fruit, spices, sweets, and condiments and beverages.

The dietary diversity score ranges from 0 to 12, where 0 means that no food was consumed at all and 12 indicates maximum dietary diversity. “There are no established cut-off points in terms of number of food groups to indicate adequate or inadequate dietary diversity for the HDDS” [22] (p. 26). Instead, dietary diversity scores should be compared across population groups and monitored over time. We have chosen to report on dietary diversity categories using two variations in the manner of classification, the first relating to the FAO Guidelines [22] and the second relating to the same South African study by Chakona and Shackleton (2018) [19] referred to in the HFIAS categorization above.

The FAO (2010) thresholds are

- Low dietary diversity: 0–3 food groups
- Medium dietary diversity: 4–5 food groups
- High dietary diversity: 6–12 food groups

Another categorization is provided by Chakona and Shackleton (2018: 5):

○ Low dietary diversity: 0–5 food groups
○ Medium dietary diversity: 6–7 food groups
○ High dietary diversity: 8–12 food groups.

#### 2.2.4. Coping Strategies Index (CSI)

Households facing constrained access to food adopt a range of behavioral responses, sometimes called coping strategies, ranging from mild to severe rationing (reducing consumption), and from borrowing to begging for food or cash (protecting consumption) [23,24]. The Coping Strategies Index (CSI) recognizes that adoption of certain strategies signifies higher levels of food insecurity, as does increasing frequency of adoption (from ‘never’ to ‘every day’). A short questionnaire with nine questions was devised (see Table 3), and positive responses are weighted by severity and frequency to calculate the CSI score for each household [25]:

1. Each coping strategy is assigned a score reflecting the number of days a week it was used—e.g., never adopted = 0; twice a week = 2; 3–6 times a week = 4.5; every day = 7.
2. Severity weights are used to multiply the score for each coping strategy—e.g., “Rely on less preferred foods” is weighted only 1, but “Skip whole days without eating” is weighted 4.
3. The scores are then summed to produce an index value for the CSI for each household, with a range of 0 to 126.

There is no definitive threshold separating food insecure from food secure households, but households can be ranked and compared, and those identified as most food insecure (i.e. with the highest CSI scores) can be prioritized for policy support.

The literature on where to set CSI score values to differentiate between food insecurity categories is scarce and the indicator manual [25] does not include guidance on how to transform the continuous variable scores into food insecurity categories. We selected two sets of thresholds used to categorize different degrees of food insecurity. The first is based on Maxwell et al. (2014) [26] reduced CSI (rCSI) cut-offs, which have been inflated to use with an expanded version of CSI scores in this study. The second set of category cut-offs is derived from a South Africa study by Drysdale et al. (2019) [27].

Maxwell and Caldwell (2008) [25] use a cut-off of rCSI = 4 to denote moderate food insecurity and a cut-off of rCSI = 10 to denote severe food insecurity. Since the rCSI has a maximum value of 56, and CSI has a maximum value of 126, the cut-off scores for CSI can be calculated as 4/56= x/126→ x =9 for moderate food insecurity and 10/56= x/126 → x = 22.5 for severe food insecurity. Our categories are defined using the following thresholds, based on Maxwell and Caldwell (2008) [25]. Food secure and mildly food insecure: CSI = 0–9

- Moderately food insecure: CSI = 10–22.5
- Severely food insecure: CSI = 23–126

Drysdale et al (2019) [27] suggest five food security categories. Our study combined some of their categories to reduce the number of possible states of food insecurity from five to three, using the following thresholds to define the categories.

- Food secure and mildly food insecure: CSI = 0–30
- Moderately food insecure: CSI = 31–60
- Severely food insecure: CSI = 61–90

The next section presents the results of our analysis for each of the four food security indicators. A panel dataset was created by appending the baseline and endline surveys and merging the monthly monitoring data and was analyzed using Stata v14.0 (StataCorp LLC, College Station, Texas, USA). Only observations with complete responses across baseline and monitoring instruments were included in the final sample (*N* = 195). Basic sample descriptives were estimated using the baseline data, which is also the source of the MAHFP estimates of self-reported hunger recalled over the previous 12 months. The remaining univariate analyses used the monitoring data to identify seasonal variations in food security. Firstly, in line with conventions established in the literature as described in the preceding section, the categorical transforms of the continuous indicator variables are used to estimate the prevalence of different levels of food insecurity by season, by food security dimension. Secondly, in response to identified limitations with the categorical approach, results using continuous variable versions of the indicators are reported: box plots showing central tendency and dispersion of the sample across indicator values and cumulative incidence curves showing the proportions of the sample reporting scores lower than values across the entire range of possible scores. Multivariate regression results are discussed thereafter: the summer and winter scores for each indicator are regressed against a set of covariates derived from the baseline data in order to identify key determinants of food insecurity, and whether they vary by season.

## 3. Results

### 3.1. Sample Descriptives

Sample characteristics are summarized in Table 4. The sample was entirely comprised of female farm workers thus there is zero variance on gender and occupation. The average age of the respondents was 37 years. Most lived in households with three other members, often a spouse or cohabiting partner and two dependants. Ten percent of the respondents had completed high school, 54 percent attained education between Grade 8 and Grade 11, 31 percent between Grade 1 and Grade 7, and five percent had no schooling.

Ten percent of respondents reported being permanently employed; the other 90 percent only worked seasonally. Thirty percent of the respondents lived on the farm where they worked; the remaining 70 percent lived in towns near to the farm. Foreign nationals comprised ~12 percent of the sample; the remaining 88 percent were South African.

The average total household expenditure among the sampled households was estimated at R2312 per month, of which R1054 was spending on food. Using the General Household Survey (GHS) 2017 [28], average household expenditure in the Northern Cape was estimated as R3,930 (SD = R3033) per month, and nationally households spent approximately R4017 (SD = R3243) per month. Although some consistency issues in the manner of questioning compromise comparability somewhat across datasets, the significantly lower spending among farm workers aligns with expectations regarding this vulnerable group.

### 3.2. Seasonality Descriptives

#### 3.2.1. MAHFP: Seasonality and Food Adequacy

Our baseline survey asked “Were there any months, in the past 12 months, in which you did not have enough food to meet your family’s needs?”. Three-quarters of the respondents (76.17 percent) reported having experienced food insecurity during the 12 months preceding the baseline survey. A follow-up question asked respondents to recall in which months they experienced food supply inadequacies.

**Figure 1:**
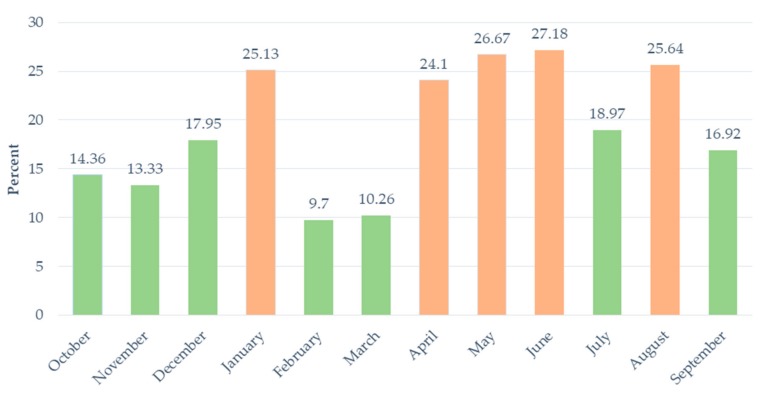
Prevalence of food inadequacy among Northern Cape farm workers by month, 2016/17. Source: FWFS study baseline survey data.

There is great variability in the prevalence of self-reported hunger during the year. Figure 1 shows that, in 2016/17, there were seven months when the prevalence of food inadequacy was relatively low (<20 percent) and five months when it was relatively high (>20 percent). February and March, the summer harvest season, recorded by far the lowest prevalence (just 10 percent of the sample). Conversely, households with inadequate food peaked during the nonfarming months of autumn and winter, with April, May, June and August all recording a similarly high prevalence (between 24 and 27 percent). The number fell below 20 percent in July, for reasons that are unclear. There was also a spike in self-reported hunger in January (25.13 percent).

#### 3.2.2. HFIAS: Seasonality and access to food

Table 5 reports on the seasonal prevalence of food insecurity using two modes of categorizing levels of food insecurity: firstly from the HFIAS Indicator Guide (Coates et al., 2007 [18]) and then from a South African study (Chakona and Shackleton, 2018 [19]).

In winter there were no households who reported being food secure, using either set of categorical thresholds. Even in the relatively high employment period during the summer harvest season, more than two out of five respondents reported severe food insecurity: the Coates et al. [18] categorization estimates 48.65 percent of the sample as severely food insecure, using the Chakona and Shackleton [19] categories the estimate is at 43.78 percent. In winter the prevalence of severe food insecurity increased to more than four out of every five households sampled: using Coates et al. [18] thresholds 87.83 percent were severely food insecure, compared to 80.42 percent using Chakona and Shackleton [19] thresholds.

Whether using Coates et al. [18] or Chakona and Shackleton [19] categories, the data shows clearly that seasonal food insecurity exists.

Whereas the seasonal trends are similarly revealed across different categorizations of food insecurity, changing the way categories are defined – whether using Coates et al. [18] or Chakona and Shackleton [19] threshold cut-offs—results in significantly different prevalence estimates, particularly in the intermediate categories of food insecurity. Chakona and Shackleton [19] categories under-report in all seasons the prevalence of moderate and severe food insecurity, and over-report mild food insecurity, compared to estimates based on Coates et al. [18] categories.

It is clear that how the HFIAS scores are translated into food insecurity prevalence categories matters greatly for how the sample is classified, i.e., choices in terms of how the indicator is treated statistically have a significant determining effect on our estimated levels of food insecurity. It is possible to avoid some of this arbitrariness by using the actual HFIAS scores.

**Figure 2:**
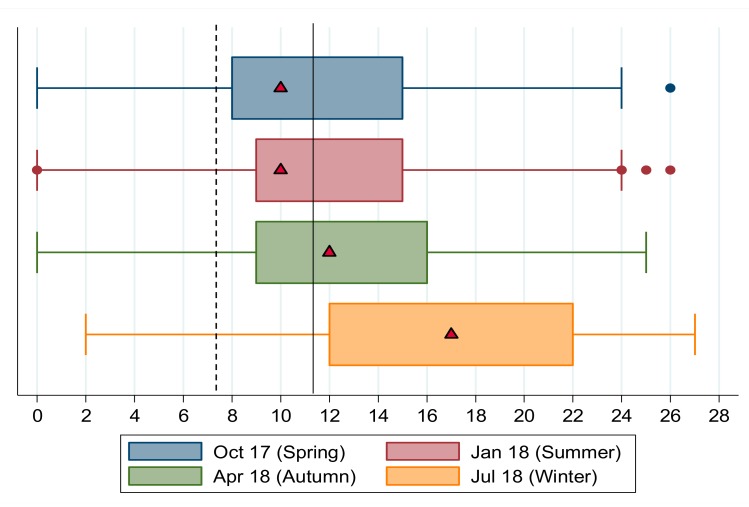
Box plots for HFIAS scores in the Northern Cape by season, 2017/18. Notes: Chakona and Shackleton’s [19] food insecurity categories are shown as vertical lines. The dashed line is the cut-off above which a household is moderately food insecure, and the solid line is the cut-off above which a household is severely food insecure. Median values shown by triangular markers. Dots represent outlier values. Source: FWFS study monitoring data.

In Figure 2, the median and interquartile ranges of HFIAS scores for each season are shown. Consistent with the categorical results and in line with expectations around our main hypothesis, the seasonality of food insecurity is clear when comparing median values of HFIAS scores across seasons. In winter the median household reported an HFIAS score of 17 compared to a summer score of 10, with spring and autumn in between, at 10 and 12, respectively. HFIAS scores are positively skewed in summer, spring and autumn; a larger share of the sample reported lower HFIAS scores, indicating lower levels of food insecurity. In winter the HFIAS distribution is negatively skewed; more households reported higher HFIAS scores and thus higher levels of food insecurity.

The vertical lines show the Chakona and Shackleton [19] cut-offs for moderate food insecurity (dashed vertical line at HFIAS = 7.5) and severe food insecurity (solid vertical line at HFIAS = 11.5). In all seasons, the median household’s HFIAS score exceeded the moderate food insecurity cut-off and in autumn and winter median scores were higher than the severe food insecurity cut-off. In winter the entire interquartile range lies above the severe food insecurity threshold, showing that more than three-quarters of the sample (in Figure 2 the exact proportion is reported at 80.42 percent) were severely food insecure.

**Figure 3:**
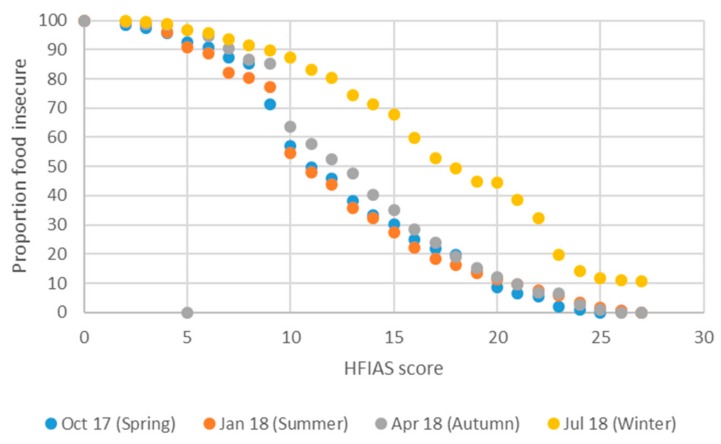
Food insecurity incidence curves for HFIAS by season, 2017/18. Source: FWFS study monitoring survey data.

The interpretation of the food insecurity incidence curves in Figure 3 is similar to the interpretation of poverty incidence curves [29], which show, generally, for different values of the poverty line the proportion of households or individuals whose income was less than the value of the poverty line, i.e., the proportion of households that are identified and classified as poor (equivalent to the poverty headcount ratio). If the poverty line is set at a very high value of income, e.g., R80,000 per household per month, then many households will be classified as poor (poverty will be over-identified). When the poverty line is set at very low levels of income, e.g., at R2500 per household per month, then much fewer households will be classified as poor (poverty will be underidentified).

Extending this logic to our food insecurity incidence curves, we see that when using the HFIAS and CSI measures (both being increasing functions of food insecurity: the higher the score the more food insecure), few people will be identified as food insecure if the score cut-off – hereafter the “food poverty line” – is set at high HFIAS/ CSI values (only those who are employing many coping strategies to deal with inadequate food, for example). The term “food poverty line” is used here to mean the value of the HFIAS (or CSI or DDS, as per the next sub-section) score against which a respondent’s score is compared in order to separate food secure from food insecure respondents. When the “food poverty line” is set at low HFIAS/ CSI values then many people are identified as food insecure (including those who only employ a few coping strategies).

Graphically, in a food insecurity incidence curve analysis, a curve that lies above other curves reflects higher levels of food insecurity. In Figure 3 the winter food insecurity incidence curve lies everywhere above all other seasons’ curves, showing unambiguously that food insecurity is worst in winter, regardless of where the food insecurity threshold is set. The summer curve lies beneath all other seasons’ curves over most of the HFIAS distribution, but crosses the spring curve in the upper tercile, where HFIAS values exceed 20.

#### 3.2.3. DDS: Seasonality and Dietary Diversity

The DDS indicator predicts that dietary diversity would be low when incomes are inadequate or irregular and high when incomes are adequate and relatively stable. Since the DDS indicator is inversely related to food insecurity, this leads to an interpretation that is opposite to that for the HFIAS and CSI indicators.

In terms of seasonality, Table 6 shows that dietary diversity was highest in summer – when most of our respondents were working and earning—using either FAO [22] cut-offs, where 95.23 percent of the sample reported high dietary diversity, or Chakona and Shackleton [19] cut-offs where almost 80 percent of the sample reported high dietary diversity. Dietary diversity was lower in winter than in summer, but lowest in autumn, when the largest shares of the sample reported the lowest levels of dietary diversity. Almost 53 percent of the sample reported low dietary diversity in autumn, compared to 4.77 percent in summer, using the Chakona and Shackleton [19] cut-offs. As noted, employment opportunities on farms for nonpermanent workers are extremely limited in autumn and winter, relative to spring and summer.

The divergence between the FAO [22] cutoff based estimates and the Chakona and Shackleton [19] based cutoffs estimates is large, for example in winter when using the FAO cut-offs over 60 percent of the sample is classified in the high dietary diversity group, compared to only a quarter of the sample being classified at the same level when using Chakona and Shackleton [19] cut-offs. With DDS, even more so than with HFIAS, using continuous indicator score values may be preferable compared to using categorical transforms of the variables, due to the very different results obtained when using slightly different score cut-offs to classify dietary diversity categories.

**Figure 4:**
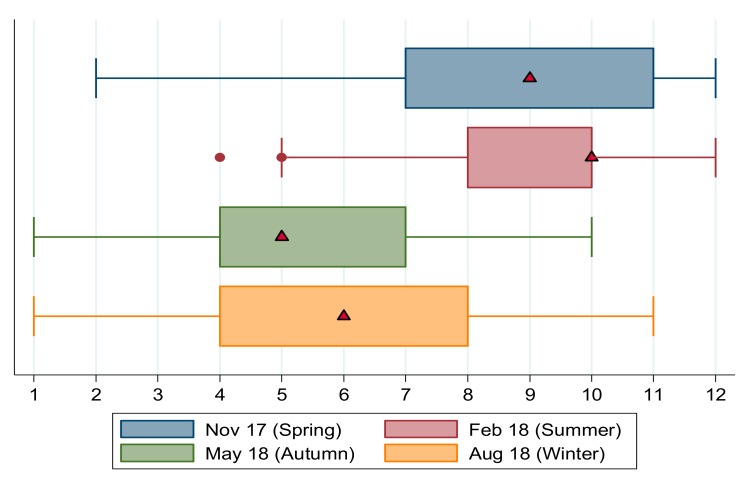
Box plots for DDS scores in the Northern Cape by season, 2017/18. Notes: Chakona and Shackleton [19] cut-offs are shown as vertical lines. The dashed line is the cut-off below which a household is moderately food insecure, and the solid line is the cut-off below which a household is severely food insecure. Median values shown by triangular markers. Dots represent outlier values. Source: FWFS study monitoring survey data.

Box plots for the continuous DDS scores are shown in Figure 4. Spring and summer are seasons of higher dietary diversity when the median number of food groups consumed was nine and 10, respectively, compared to autumn and winter when respondents reported eating from only five and six food types, respectively.

The thresholds used by Chakona and Shackleton [19] are depicted as a solid vertical line, below which households are classified as having low dietary diversity (severe food insecurity) and a dashed vertical line at DDS = 7.5, denoting the upper bound cut-off defining medium dietary diversity (moderate food insecurity). In autumn the median DDS lies to the left of both the medium and low dietary diversity cut-offs indicating low levels of food security. In winter the median household’s dietary diversity lies toward the lower bound of the moderate food insecurity category, with most of the second quartile’s DDS scores below the severe food insecurity threshold. In summer and spring, the median household consumed a diverse diet, with scores higher than both food insecurity cut-offs. This shows bimodality in the distribution of DDS scores, that is, clear differences in food security across seasons, with spring and summer scores significantly higher than autumn and winter scores.

**Figure 5:**
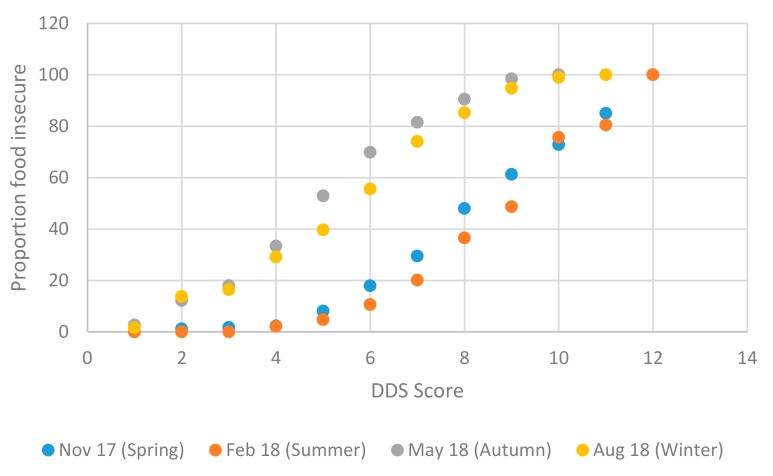
Food insecurity incidence curves for Dietary Diversity Scores (DDS) by season, 2017/18. Source: FWFS study monitoring survey data.

This bimodal distribution of scores across seasons is shown clearly in the food insecurity incidence curves in Figure 5. Respondents with low DDS scores consumed limited types of foods; in contrast to HFIAS scores which have an inverse relationship with food security, DDS scores are positively related to food security. For this reason, DDS based food insecurity incidence curves slope upward, in contrast to HFIAS curves.

Our data partially reflects seasonality expectations: the summer curve lies, for the majority of the distribution, beneath all the other seasons’ curves, showing higher levels of dietary diversity during this period. The winter curve, however, lies beneath the autumn curve for most of the DDS score distribution, except for in the tails. Despite the ambiguous results between winter and autumn, there is an unambiguous bimodality in the cumulative distribution, showing that dietary diversity and hence food insecurity are worse in autumn and winter than in spring and summer.

#### 3.2.4. CSI: Seasonality and Coping Strategies

Table 7 reports estimates of prevalence of food insecurity using the Maxwell et al. (2014) [26] based and Drysdale et al. (2019) [27] based thresholds to classify respondents into different groups of varying food insecurity.

Estimates of the prevalence of food insecurity vary drastically depending on whether Maxwell et al.’s [26] cut-offs or Drysdale et al.’s [27] cut-offs are used, with Drysdale et al. [27] thresholds leading to consistent under-reporting of food insecurity relative to the Maxwell et al. [26] cut-offs. The levels of severe food insecurity are higher in winter compared to other seasons. Using Maxwell et al.’s [26] cut-offs, about two-thirds of the sample is classified as severely food insecure in winter, compared to 37 percent in autumn and 46.48 percent in summer. The pattern of food insecurity between autumn and summer is ambiguous.

**Figure 6:**
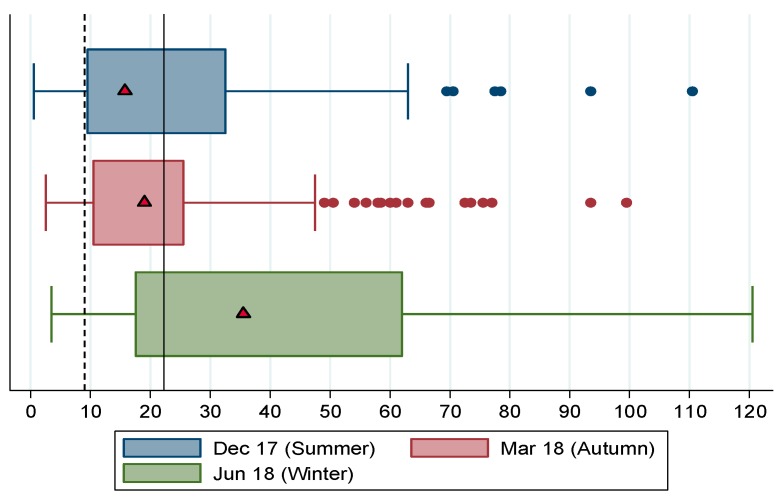
Box plots for CSI scores in the Northern Cape by season, 2017/18. Notes: Maxwell et al.’s [26] food insecurity category thresholds shown as vertical cut-offs. The dashed vertical line is the cut-off above which the household is moderately food insecure and the solid vertical is the cut-off above which the household is severely food insecure. Median values shown by triangular markers. Dots represent outlier values. Source: FWFS study monitoring survey data.

Figure 6 shows the box plots for CSI over summer, autumn and winter. The distribution of CSI scores across seasons show unambiguously that food security is worse in winter, when households used more coping strategies and more frequently (median score of 35.5) compared to summer (median = 15.75) and autumn (median = 19). In all seasons the median household’s CSI score exceeds the cut-off for moderate food insecurity, that is, in all seasons the median household was at least moderately food insecure, and in winter the median household was severely food insecure.

**Figure 7:**
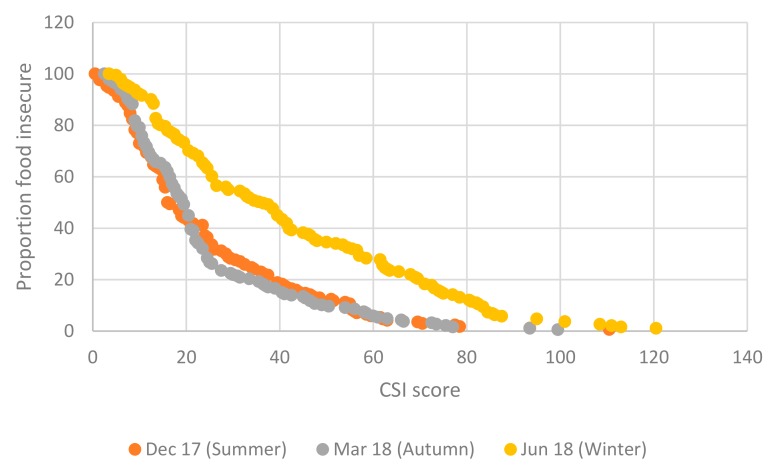
Food insecurity incidence curves for CSI by season, 2017/18. Source: FWFS study monitoring survey data.

Some clarity on the ambiguity between autumn and summer CSI based identifications and ranking of food insecurity is gained from the box plots, in terms of the autumn median exceeding the summer median (showing that food insecurity was worse in autumn than in summer). The ambiguity is further illustrated by means of the CSI food insecurity incidence curves shown in Figure 7, which show that up to a CSI score of 20, and again at CSI scores above 58.5, the summer incidence curve lies below the autumn curve, indicating lower food insecurity in summer than autumn. In the intermediate range of CSI scores (over 20 and under 58.5) the autumn curve lies beneath the summer curve. The crossing of the summer and autumn incidence curves suggest that rankings of food insecurity by season using CSI will be highly sensitive to the level at which the threshold for categorical identification is set. What is unambiguous with respect to the incidence curves is that, in line with our expectations, the winter incidence curves lies everywhere above the autumn and summer curves, showing that food insecurity is worst in winter across the entire range of CSI scores.

### 3.3. Multivariate Analysis: Correlates of HFIAS, DDS, and CSI Indices

Table 8 shows results from selected multivariate regressions for summer and winter for each indicator. All models use the actual score values for each of the food insecurity indicators, thus Ordinary Least Squares regression can be used. The models presented for each indicator for each season include a constrained list of covariates. Only those that were significant in at least one of the models were included. Some variables have been excluded because running the model with them produced tiny and insignificant coefficient estimates. The final explanatory variables included are the number of years of schooling, household size, whether the respondent is a seasonal or permanent worker, whether they live off or on the farm, whether they are a South African or a foreign national and their total monthly household expenditure (including food expenditure).

The overall explanatory power of the models is low: the model which accounts for most variance in food insecurity is for the summer CSI scores (R^2^ = 0.233). The models for DDS perform worst, with R^2^s ranging from 0.071 to 0.96 for summer and winter models. That the models only explain a small part of the overall variance in food insecurity is not unexpected given the limited number of covariates captured in the survey, limited variance in the characteristics of the respondents and a small sample size. These factors likely also account for the limited statistical significance of the coefficient estimates.

*Years of Schooling*

Our expectations are that an additional year of schooling will reduce food insecurity, so for HFIAS and CSI we expect negative coefficients, and for the DDS models we expect positive coefficients. The results shown in Table 8 conform to these expectations, even though only the CSI model estimates are statistically significant at conventional levels of significance. A one year increase in schooling leads to a decrease of 1.097 CSI points in summer and 1.680 CSI points in winter, ceteris paribus.

*Household Size*

In the rest of Africa, increasing household size is related to higher poverty levels due to higher dependency ratios. In South Africa, the effect of increasing household size does not necessarily follow this pattern, partly due to the presence of social grants: millions of older persons and children in poor households receive a relatively generous Older Persons Grant or Child Support Grant from the state every month. Our results suggest an indeterminate effect of household size on food insecurity. Of the results that are statistically significant at all conventional levels of significance, the HFIAS summer model suggests that an additional household member reduces the HFIAS score by 0.497 points ceteris paribus whereas the winter CSI model suggests that an additional household member increases the number of coping strategies employed by the household by 3.205 points on the CSI indicator ceteris paribus.

*Seasonal vs. Permanent Worker*

We expect that being a seasonal worker compared to a permanent worker is associated with lower levels of food security, given that employment and earnings are less secure. These expectations are not borne out by the HFIAS models, which suggests a positive effect of seasonality on food insecurity, although these estimates are not statistically significant at any conventional levels of significance. The only model where the seasonal worker coefficient is statistically significant is for DDS in winter, where being a seasonal worker compared to being permanently employed is associated with a reduction of 1.789 food types (or DDS points).

*Lives on Farm vs. off Farm*

Only the HFIAS winter model and the two DDS models are significant for the variable capturing whether the worker lives on or off the farm. Living off the farm is associated with a reduction in HFIAS by 2.864 points, showing that living off the farm where employed is associated with lower food insecurity, possibly because living in town offers access to more sources of food and income. By contrast, the effect of living off the farm is to reduce dietary diversity by almost one food group in winter, but to increase it by one food group in summer.

*South African vs. Foreign National*

For all models, being South African compared to foreign national is associated with lower levels of food security in all seasons. Being South African increases the HFIAS score by 3.5 points in summer and 5 points in winter ceteris paribus, and this result is significantly better than the 95 percent level of significance. The effects are insignificant for DDS. For CSI, being South African is associated with using more coping strategies; in summer the CSI increases by 11.33 points and in winter the increase is even larger, at 24.26 points.

*Total Household Expenditure*

Income and spending are expected to have a significant positive impact on household food security. A one Rand increase in monthly household expenditure is associated with a reduction in the HFIAS score by 3.395 points in summer and 2.39 points in winter ceteris paribus, and this result is statistically significant at better than the 1 percent level. For DDS the results are not significant at any conventional levels but in both summer and winter a one Rand increase in household expenditure is associated with an increase in dietary diversity. The CSI model results are contradictory across seasons: in summer a one Rand increase in household spending is associated with a 9.22 point reduction in the CSI index (significant at the 0.1 percent level of significance). In winter, the coefficient changes sign, however it is not significant.

## 4. Discussion

This research study aimed to test the hypothesis that seasonal hunger exists among farm workers in South Africa, not—as elsewhere in Africa—through intra-annual fluctuations in food availability due to poor farming families’ reliance on a single main annual harvest [1,2,5,6], but through intra-annual fluctuations in employment opportunities on commercial farms. The specific pathway to hunger was identified as lack of employment in the nonfarming season, resulting in inadequate income to buy sufficient food to meet household consumption needs during the winter months.

There is no consensus in the literature on the ‘best’ indicator of household food security [26,30]. Instead of choosing one indicator to test our hypothesis we collected data on four complementary indicators [16,18,20,25], which allows us to compare and triangulate the findings. These indicators have different recall periods, ranging from 24 hours to one year, and they offer different perspectives on food insecurity, from types of food consumed to subjective experiences of hunger, to ‘coping strategies’ for dealing with food insecurity.

Because each indicator measures different aspects of food insecurity, we did not expect the four indicators to produce similar estimates of percentages of food insecure people in absolute terms. The proportion of households with low dietary diversity is not directly comparable to the proportion of households deploying a high number of coping strategies, for instance. But we did expect that each indicator would follow a similar trajectory through the year, and that the shape of this trajectory would be consistent across the indicators. Specifically, we expected all four indicators to reveal relatively high levels of food insecurity during winter, relatively low levels during summer, and intermediate levels during spring and autumn.

Our findings broadly confirm this pattern, which is displayed fairly consistently for each separate indicator and across the four indicators. There is robust evidence of seasonal fluctuations in food (in)security among farm worker households in the Northern Cape province of South Africa. Summer months are relatively food secure, with better access to employment, income and food. Winter months are hard, with little or no employment opportunities, low or zero income—many families survive on social grants from governments targeted at poor children and older persons [8,19]—and restricted access to food. Follow-up qualitative research is planned, partly to explain some of the incongruous findings reported in this article, such as the anomalous finding for MAHFP in July, and inconsistencies across seasons for DDS and CSI.

How do our findings compare to other relevant estimates of food insecurity in South Africa? The national annual General Household Survey (GHS) includes a question eliciting self-reported hunger among adult household members. In the 2017 GHS, 83.09 percent of South African adults reported never having experienced hunger during the 12 months prior to the survey, but adults in the Northern Cape were worse off than in any other province: only 75.77 percent reported that they had experienced no hunger during the reference period, and those in rural Northern Cape were even worse off with just 66.14 percent reporting no hunger [28]. This has implications for the interpretation and contextualization of our results. Given that our sample is comprised entirely of Northern Cape female farm workers—one of the poorest groups living in rural areas of the most food insecure province of South Africa—we might expect their food security status to be even worse than the GHS estimate for rural Northern Cape. This is confirmed in our baseline survey, when 76.16 percent of respondents self-reported that they had experienced hunger in the 12 months preceding the survey.

Very few South African studies have measured self-reported household food security using HFIAS, DDS or CSI. Chakona and Shackleton (2018) [19] estimated HFIAS for a sample of 554 women from low-income households in KwaZulu-Natal province. Averaged over pre- and postharvest seasons, 12 percent of their sample was classified as severely food insecure, 28 percent as moderately food insecure, 24 percent as mildly food insecure, and 36 percent as food secure [19]. This contrasts with our findings from Northern Cape farm workers, where severe food insecurity as measured by HFIAS ranged from 43.78 percent in summer to 80.42 percent in winter, and less than 2 percent of our sample reported being food secure in any season. One possible factor explaining the lower prevalence of food insecurity in KwaZulu-Natal could be that Chakona and Shackleton’s [19] sample was drawn from three towns and included urban and peri-urban as well as rural residents.

The Chakona and Shackleton (2018) [19] study is a useful comparator for our DDS data. Their combined sample share estimates report 6 percent with low dietary diversity, 31 percent with medium, and 63 percent with high dietary diversity. Our figures are congruent with theirs during spring and summer in terms of having smallest shares in low dietary diversity (8.09 percent in spring and 4.77 percent in summer) and largest shares with highest dietary diversity (70.52 percent during spring and 79.89 percent in summer). During winter and autumn, however, most of our sample do not attain high levels of dietary diversity.

Our CSI based food security categories were constructed using the same categories as Drysdale et al. (2019) [27]. But Drysdale et al. [27] do not report prevalence estimates. However, Kruger et al. (2008) [31] explored the “food coping strategies” of 13 female farm workers living on one farm in Free State province [31]. Because of the small sample size classification into food insecurity categories was not possible, but the Coping Strategies Index (CSI) methodology [25] was adapted to estimate the severity of food stress across five seasons. Taking a CSI score of 55 as a cut-off, Kruger et al. [31] found their sample as a whole to be food secure (CSI < 55) in spring, early summer and autumn, but food insecure (CSI > 55) during late summer and winter. The increase in food stress in late summer (December–January) “is the opposite of what is expected” [31] (p. 11), but it coincides with our own observed spike in food insecurity in January (Figure 1 above).

## 5. Limitations

Two key limitations arise from the sampling method. Firstly, given that participants were not randomly selected into the sample, the results have low external validity and cannot be generalized to any larger population. Secondly, the small sample size and the lack of variance in sample characteristics means that any analysis of the determinants of food security is constrained, and only a small proportion of the observed variance in food security can be explained by variance in characteristics of the sample.

The self-reported measures of food insecurity used in this study may present another limitation. Although some studies have found statistically significant and proportionate relationships between self-reported food security and actual dietary intake [32], and between self-reported food security and anthropometric measures of nutrition [33], others have argued that self-reported measures tend to overstate food insecurity [34].

## 6. Conclusions

Seasonal hunger has been recorded in poor rural communities in many African countries, as noted earlier [2,4,5,6]. But in other countries seasonal hunger affects mainly smallholder farmers, whereas in South Africa the worst affected group is commercial farm workers. South Africa is an upper-middle income country, and most of its residents do not experience food insecurity for several months each year. In this context, our findings are shocking. There are clear implications for social and economic policy. More attention must be paid to the vulnerability of farm workers, and solutions must urgently be found to address their ‘hidden crisis’ of underemployment and seasonal hunger.

## Figures and Tables

**Table 1:** Indicators of household food insecurity collected.

Year	2017				2018									
Season	Spring			Summer			Autumn			Winter			Spring	
Indicator	Sep	Oct	Nov	Dec	Jan	Feb	Mar	Apr	May	Jun	Jul	Aug	Sep	Oct
MAHFP														
HFIAS														
DDI														
CSI														

Source: Farm Worker Food Security (FWFS) study planning documentation. Note: The indicators include Months of Adequate Household Food Provisioning (MAHFP), Household Food Insecurity Access Scale (HFIAS), Dietary Diversity Index (DDI) and Coping Strategies Index (CSI). Grey boxes show when each indicator survey was administered CSI data not collected for September 2018 (shown by black box).

**Table 2:** Household Food Insecurity Access Scale (HFIAS) questionnaire.

1	In the past 4 weeks, did you worry that your household would not have enough food?
2	In the past 4 weeks, were you or any household member not able to eat the kinds of foods you preferred, because of a lack of resources?
3	In the past 4 weeks, did you or any household member have to eat a limited variety of foods, due to a lack of resources?
4	In the past 4 weeks, did you or any household member have to eat some foods that you really did not want to eat, because of a lack of resources to obtain other types of food?
5	In the past 4 weeks, did you or any household member have to eat a smaller meal than you felt you needed, because there was not enough food?
6	In the past 4 weeks, did you or any household member have to eat fewer meals in a day, because there was not enough food?
7	In the past 4 weeks, was there ever no food to eat of any kind in your household, because of lack of resources to get food?
8	In the past 4 weeks, did you or any household member go to sleep at night hungry, because there was not enough food?
9	In the past 4 weeks, did you or any household member go a whole day and night without eating anything, because there was not enough food?

Source: Coates, Swindale, and Bilinsky (2007: 5).

**Table 3:** Coping Strategies Index (CSI) questionnaire.

**In the past month, how often have you had to**
1. Rely on less preferred and less expensive food?
2. Borrow food, or borrow money to buy food?
3. Purchase food on credit?
4. Rely on help from relative or friend outside household?
5. Limit portions at meal-times?
6. Ration the little money you have to household members to buy street foods?
7. Limit your own intake to ensure child gets enough?
8. Reduce number of meals eaten in a day?
9. Skip whole days without eating?

Source: adapted from Maxwell and Caldwell, 2008 [25].

**Table 4:** Sample characteristics.

	Mean (SD)/Proportion
Age	37 years (11)
Education	
No schooling	0.05
Grade 1–7	0.31
Grade 8–11	0.54
Grade 12	0.10
Household size	4 (2)
Employment type	
Seasonal	0.90
Permanent	0.10
On or off farm residence	
Lives off farm	0.70
Lives on farm	0.30
Years working on farm	
5 years or less	0.51
6 to 11 years	0.22
11 to 19 years	0.17
20 years or more	0.1
Nationality	
South African	0.88
Foreign national	0.12
Monthly household food expenditure	R1054 (R686)
Total monthly household expenditure	R2312 (R1555)

Source: FWFS study baseline survey data (2017). Notes: *N* = 195, Rand in 2017 prices, where means are reported Standard Deviation (SD) in brackets.

**Table 5:** Proportions of sample classified in each food security category: HFIAS.

Period	HFIA Categories	Proportion of Sample (%)	
		Coates et al. (2007)* [18] Categories	Chakona and Shackleton (2018) [19] Cut-offs
Oct 2017 (Spring)	Food secure ^a^	1.59	1.59
	Mildly food insecure ^b^	1.59	13.23
	Moderately food insecure ^c^	30.69	39.15
	Severely food insecure ^d^	66.14	46.03
Jan 2018 (Summer)	Food secure ^a^	0.54	0.54
	Mildly food insecure ^b^	3.78	18.92
	Moderately food insecure ^c^	47.03	36.76
	Severely food insecure ^d^	48.65	43.78
Apr 2018 (Autumn)	Food secure ^a^	0.53	0.53
	Mildly food insecure ^b^	1.06	12.70
	Moderately food insecure ^c^	37.77	34.39
	Severely food insecure ^d^	60.64	52.38
Jul 2018 (Winter)	Food secure ^a^	0.00	0.00
	Mildly food insecure ^b^	0.53	8.47
	Moderately food insecure ^c^	11.64	11.11
	Severely food insecure ^d^	87.83	80.42

Source: FWFS study monitoring survey data. Notes: * Categories generated as per Coates et al. [18] described in Section 2.2.2 above. Chakona and Shackleton [19] categories use the following cutoffs for HFIAS score: ^a^ 0–1, ^b^ 2–7, ^c^ 8–11, ^d^ 12–27.

**Table 6:** Proportions of sample classified in each dietary diversity category: Dietary Diversity Score (DDS).

Period	Categories of Dietary Diversity	Proportion of Sample (%)	
		FAO (2010) [22] Cut-offs	Chakona and Shackleton (2018) [19] Cut-offs
Nov 2017 (Spring)	Low dietary diversity ^a,d^	1.73	8.09
	Medium dietary diversity ^b,e^	6.36	21.39
	High dietary diversity ^c,f^	91.91	70.52
Feb 2018 (Summer)	Low dietary diversity ^a,d^	0	4.77
	Medium dietary diversity ^b,e^	4.77	15.34
	High dietary diversity ^c,f^	95.23	79.89
May 18 (Autumn)	Low dietary diversity ^a,d^	17.99	52.91
	Medium dietary diversity ^b,e^	34.92	28.57
	High dietary diversity ^c,f^	47.09	18.52
Aug 18 (Winter)	Low dietary diversity ^a,d^	16.41	39.69
	Medium dietary diversity ^b,e^	23.28	34.39
	High dietary diversity ^c,f^	60.31	25.92

Source: FWFS study monitoring survey data. Notes: FAO [22] cutoffs for DDS scores are ^a^ 0–3, ^b^ 4–5, ^c^ 6–12 and Chakona and Shackleton [19] score cutoffs are ^d^ 0–5, ^e^ 6–7, ^f^ 8–12.

**Table 7:** Proportions of sample classified in each food security category: CSI.

Period		Proportion of Sample (%)	
		Maxwell et al. (2014) [26] Based Cut-offs	Drysdale et al. (2019) [27] Based Cut-offs
Dec 2017 (Summer) *n* = 196	Food secure and mildly food insecure ^a,d^	19.90	62.75
	Moderately food insecure ^b,e^	31.12	18.88
	Severely food insecure ^c,f^	46.48	18.37
Mar 2018 (Autumn) *n* = 191	Food secure ^a,d^	19.95	75.00
	Moderately food insecure ^b,e^	43.01	15.00
	Severely food insecure ^c,f^	37.04	10.00
June 2018 (Winter) *n* = 196	Food secure ^a,d^	7.14	45.54
	Moderately food insecure ^b,e^	26.02	26.70
	Severely food insecure ^c,f^	66.84	30.76

Source: FWFS study monitoring survey data. Notes: The Maxwell et al. [26] cutoffs for CSI scores ^a^ 0–9, ^b^ 10–22.5, ^c^ >23 and the Drysdale et al. [27] score cutoffs are ^d^ 0–30, ^e^ 31–60, ^f^ 61–90.

**Table 8:** Correlates of food insecurity: comparison of summer and winter HFIAS, DDS, and CSI.

	HFIAS Summer	HFIAS Winter	DDS Summer	DDS Winter	CSI Summer	CSI Winter
	b (p)	b (p)	b (p)	b (p)	b (p)	b (p)
yrschooling	−0.257 (0.05)	−0.208 (0.19)	0.069 (0.21)	−0.035 (0.58)	−1.097 * (0.03)	−1.680 * (0.02)
HHsize	0.497 ** (0.01)	−0.445 (0.06)	0.112 (0.16)	0.015 (0.86)	0.850 (0.24)	−3.205 ** (0.00)
seasonal	−0.400 (0.76)	−2.562 (0.12)	−0.539 (0.32)	−1.789 ** (0.00)	7.631 (0.12)	−4.474 (0.52)
off_farm	−0.451 (0.57)	−2.864 *** (0.00)	−0.923 ** (0.01)	1.080 ** (0.00)	−5.589 (0.08)	2.977 (0.49)
SAfrican	3.498 ** (0.00)	5.003 *** (0.00)	−0.352 (0.46)	−0.235 (0.66)	11.334 * (0.02)	24.269 *** (0.00)
logtotexp	−3.395 *** (0.00)	−2.390 ** (0.00)	0.157 (0.52)	−0.460 (0.11)	−9.224 *** (0.00)	4.052 (0.22)
constant	34.887 *** (0.00)	38.572 *** (0.00)	8.413 *** (0.00)	10.641 *** (0.00)	85.979 *** (0.00)	18.133 (0.53)
r^2^	0.233	0.188	0.071	0.096	0.211	0.157
df_r	173.000	177.000	177.000	177.000	158.000	179.000

Source: FWFS study baseline and monthly monitoring survey data. Notes: *p*-values reported in parenthesis. * *p* < 0.05, ** *p* < 0.01, *** *p* < 0.001.
